# Complex evolution of the *GSTM* gene family involves sharing of *GSTM1* deletion polymorphism in humans and chimpanzees

**DOI:** 10.1186/s12864-018-4676-z

**Published:** 2018-04-25

**Authors:** M. Saitou, Y. Satta, O. Gokcumen, T. Ishida

**Affiliations:** 1 Department of Biological Sciences, The University of Tokyo, Tokyo, Japan; 2 Department of Biological Sciences, State University of New York at Buffalo, Buffalo, USA; 3 The Graduate University for Advanced Studies (SOKENDAI), Hayama, Japan

**Keywords:** Copy number variation, Structural variants, Detoxifying gene family, Primates, Gene conversions, Segmental duplications

## Abstract

**Background:**

The common deletion of the glutathione S-transferase Mu 1 (*GSTM1*) gene in humans has been shown to be involved in xenobiotic metabolism and associated with bladder cancer. However, the evolution of this deletion has not been investigated.

**Results:**

In this study, we conducted comparative analyses of primate genomes. We demonstrated that the *GSTM* gene family has evolved through multiple structural variations, involving gene duplications, losses, large inversions and gene conversions. We further showed experimentally that the *GSTM1* was polymorphically deleted in both humans and also in chimpanzees, through independent deletion events. To generalize our results, we searched for genic deletions that are polymorphic in both humans and chimpanzees. Consequently, we found only two such deletions among the thousands that we have searched, one of them being the *GSTM1* deletion and the other surprisingly being another metabolizing gene, the *UGT2B17*.

**Conclusions:**

Overall, our results support the emerging notion that metabolizing gene families, such as the *GSTM, NAT, UGT* and *CYP*, have been evolving rapidly through gene duplication and deletion events in primates, leading to complex structural variation within and among species with unknown evolutionary consequences.

**Electronic supplementary material:**

The online version of this article (10.1186/s12864-018-4676-z) contains supplementary material, which is available to authorized users.

## Background

The majority of variable base pairs among human genomes are due to structural variation, *i.e.*, relative deletions, duplications, inversions and translocations of segments of DNA [1–4]. For example, among 2504 individuals, the cumulative number of variable base pairs due to single nucleotide variants (as compared to the reference genome) is 33.8Mbp, roughly corresponding to 1% of the human reference genome. In contrast, structural variants cumulatively cover ~ 217Mbp (~ 7%) of the reference genome, with deletions and duplications covering ~ 2.8% and 4.4% of the genome, respectively [5]. Despite the fact that the overall genomic impact of structural variants is now appreciated, their functional impact remains largely unknown.

It is assumed that structural variants can have a profound functional impact when they overlap with coding sequences. For example, a large, complete deletion of a protein coding gene will obviously lead to the elimination of the expression of that protein. As a consequence, most of the large deletion polymorphisms (*i.e.*, kilobase level events, and not smaller insertion-deletion polymorphisms) that are common in human populations are depleted for coding sequences [5]. Complete gene deletions are even rarer [5]. As such, the deletion of the glutathione S-transferase mu 1 (*GSTM1*) gene is an unusual case, as it reaches major allele status in most human populations (*e.g.*, ~ 70% in Eurasian populations) [6–12]. This deletion variant has been associated strongly with susceptibility to bladder cancer [13], multiple sclerosis [14] and early onset of severe mental disorders [15], among other diseases. However, why this deletion has been maintained in human populations remains unknown.

**Fig. 1:**
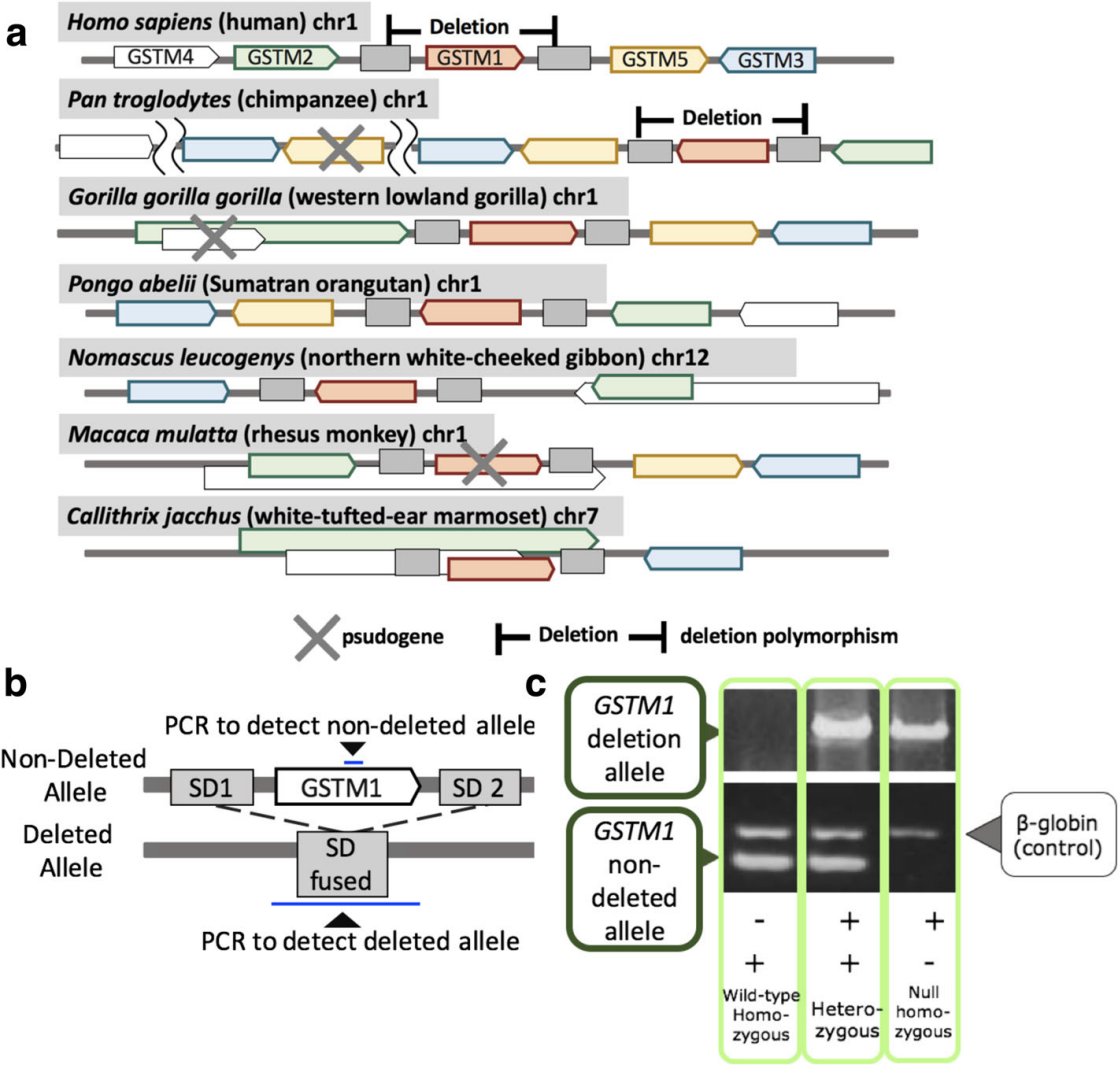
The *GSTM* locus evolution: **a** The chromosomal locations of primate *GSTM* genes and the segmental duplication sequences confirmed by tblastn and NCBI gene annotations (https://www.ncbi.nlm.nih.gov/gene). Colors indicate each of the *GSTM* orthologs based on sequence similarity (Additional file 1: Figure S1AB). Diamond indicates deletion polymorphism. Cross indicates pseudogene. Note that there may be some annotation issues with regards to some of the genes, especially outside of the great ape lineages. For example, the *GSTM2* and *GSTM4* in the gibbon as reported in NCBI overlap with each other. These overlapping genes are indicated by boxes on top of each other. **b** The location of primers and (**c**). Genotyping methods for the *GSTM1*. The *GSTM1* non-deleted allele fragment was 215 bp and the β-globin fragment as internal control was 268 bp. The *GSTM1* deletion-allele fragment was about 12kbp, enpassing the fused SD. Individuals with only the *GSTM1* non-deleted allele were considered as non-deleted homozygous, individuals with both the *GSTM1* non-deleted type allele and deletion allele were considered as heterozygous and individuals with the *GSTM1* deletion allele were considered as deletion homozygous genotype. Primers are described in Additional file 1: Table S3

The *GSTM1* belongs to the large *GST* gene superfamily. All of the dozens of different proteins coded by *GST* genes are involved in the metabolic detoxification of products generated by oxidative stress, electrophilic compounds, carcinogens, environmental toxins and therapeutic drugs [16]. Their functional location range from mitochondria, membrane-bound to cytosolic (reviewed in [17]). In the human reference genome, there are seven GST gene families, each likely formed by gene duplications, and thus similar to each other in sequence and chromosomal location [18]*.* The GST-μ (*GSTM*) family, to which the *GSTM1* belongs, is comprised of five highly similar (*e.g.*, the *GSTM1* coding sequence is 90% identical to the *GSTM2*) tandem *GSTM* genes on the chromosome 1 in humans (Fig. 1a) [19].

Metabolizing genes, such as the members of the *GSTM* family, have been reported on several occasions to harbor adaptive single nucleotide and structural variation. For example, cytochrome P450 2D (*CYP2D*) gene family underwent frequent gene duplications, losses and gene conversions in primates with implications to drug metabolism variation in humans [20, 21]. More focused studies have shown that human CYP2D6 enzyme metabolizes about 25% of commonly used drugs [22]. Moreover, the variation in different *CYP* gene family members have been reported to evolve under non-neutral forces in humans, possibly as a response to variation in dietary intakes, such as salt consumption [23, 24]. Similar to *CYP* genes, *NAT2* (N-acetyltransferase 2) has also been reported to be evolving under non-neutral conditions in non-human primates, as well as among human populations [20, 21, 25]. Deletion of another metabolizing gene *UGT2B17* (uridine diphosphoglucuronosyltransferase) has been shown to adaptively increase in frequency in East-Asian populations [26]. Last but not least, in the context of human evolution, Lin et al. [27] found that a gene deletion of another member of the *GST* superfamily, *GSTT1*, was unusually old and showed signatures of balancing selection. The diverse evolutionary trajectories of metabolizing genes are often attributed to changes in dietary xenobiotic exposure during evolutionary time [28], as well as geography-specific abundances of specific toxic substances [29].

The *GSTM* gene family varies in composition among primates with unknown functional consequences. For example, a locus-specific study has shown that the *GSTM1* was not expressed as a functional gene in a cynomolgus macaque (*Macaca fascicularis*) [30]. Rapid change in the number and type of gene families to fine-tune the functional repertoire has been shown, especially within the context of host-pathogen arms race [31]. In addition, it is important to note here that several studies have shown loss-of-function variations can underwend positive selection in primates [32]. Overall, it is plausible that the common polymorphic deletion of the *GSTM1* gene, may have been have been evolving under non-neutral conditions.

The deletion of the *GSTM1* gene has likely been facilitated by the architecture of the *GSTM* locus. The *GSTM* family members were generated by multiple segmental duplications, which are near identical segments of DNA larger than 1 kb [6]. These segmental duplications construct a critical architectural feature that may help explain the mechanism through which the *GSTM1* deletion has formed. Specifically, segmental duplications tend to cause gene duplication and deletion events by facilitating non-allelic homologous recombinations [33]. Consequently, they are the main underlying genomic feature that has contributed to the evolution of gene families in primates [34]. In fact, previous studies have shown that gene deletions and duplications in such complex regions are major contributors to evolutionary innovation [34–36]. For the *GSTM* locus, Uno et al. [30] reported that the duplicated nature of the region is similar between humans and macaques. It is likely that deletion of *GSTM1* in humans is a result of non-allelic homologous recombination event facilitated by two segmental duplications flanking this gene in the primate genomes [6]. It is also possible that other lineage-specific gene duplications or deletions may have occurred in other primates. However, there is no a systematic study to document such events. Therefore, in this study, we investigated the variation in the *GSTM* locus among primates and specifically the origins of the *GSTM1* gene deletion in humans.

## Results

### *GSTM* locus has evolved through multiple structural variants in primates

To fully understand the evolutionary context of the *GSTM1* deletion, we first conducted in silico comparative genomic analyses among primates (see methods). Based on our analyses, we found that great ape genomes carry 4–6 functional *GSTM* genes and also a varying number of lineage-specific pseudogenes (Table 1). Our phylogenetic analyses of the different *GSTM* genes among primates (Additional file 1: Figure S1A and B) indicated that all five *GSTM* genes in humans have emerged before great ape and Old World monkey lineages diverged from each other. Based on both maximum likelihood and neighbor-joining approaches, we also conclude that the *GSTM3* diverged from other *GSTM* genes early in the evolution of this gene family. Further scrutiny of the locus revealed that both copy number, location and direction of individual *GSTM1* genes are shuffled within the same locus (Fig. 1a, Additional file 1: Figure S2). For example, the chimpanzee *GSTM* locus differs from that of humans by a large inversion event encompassing the *GSTM1, GSTM2, GSTM3,* and *GSTM5*, as well as a duplication encompassing the *GSTM5* and the *GSTM3.* It is also of note that the duplicated *GSTM5* in chimpanzees has gained loss-of-function variants, and hence become a pseudogene.

**Table 1:** The number of *GSTM* genes found in each species

Species	Functional genes	Pseudogenes
*Homo sapiens*	5	1
*Pan troglodytes*	5	5
*Pan paniscus*	6	1
*Gorilla gorilla gorilla*	4	1
*Pongo abelii*	5	0
*Nomascus leucogenys*	4	2
*Macaca mulatta*	4	3
*Callithrix jacchus*	4	3
*Tupaia belangeri chinensis*	2	3

The result of these complex events led to different repertoires of the *GSTM* genes even among closely related primate species. For example, there seems to be an additional active *GSTM3* gene in chimpanzees as compared to humans, while gorilla appears to be missing *GSTM4*. The orang-utan genome, even though harboring a similar number of *GSTM* genes with humans, show remarkable difference in the direction and relative location of these genes as compared to the human genome. Overall, our results support the notion that the *GSTM* region in great apes, and likely in all primates, is rapidly evolving through lineage-specific duplication, deletion, inversion and pseudogenization events.

### The *GSTM1* is polymorphically deleted both in humans and chimpanzees

Next, we extensively investigated the presence of a *GSTM1* deletion in chimpanzees. We reasoned that the *GSTM1* may be prone to non-allelic homologous recombination in chimpanzees due to the segmental duplications flanking the *GSTM1* as has happened for humans. Specifically, we conducted polymerase chain reaction based amplification to genotype a putative chimpanzee deletion, using primer sequences modified from those primers previously used to genotype the human *GSTM1* deletion [37, 38] (Fig. 1b and c). We found that the *GSTM1* is also commonly deleted within chimpanzees. Specifically, out of the 37 chimpanzees, we found 6 and 17 of them to carry homozygous and heterozygous deletions of the *GSTM1*, respectively. The deletion allele frequency was thus 0.41 (29/74). We confirmed the presence of the chimpanzee polymorphic deletion using Droplet Digital PCR (ddPCR, Bio-Rad, Hercules, USA) and read-depth methods in 4 chimpanzee samples used in a previous study [39] (Additional file 1: Figure S3).

To explain what maintained the *GSTM1* deletion polymorphic both in humans and chimpanzees, we considered two scenarios. First, it is plausible that the deletions observed in humans and chimpanzees are identical by descent and have remained in both human and chimpanzee populations due to incomplete lineage sorting. Such allele sharing in functional sequences has often been discussed within the context of balancing selection [40–42]. Second, it is also plausible that the deletion has occurred recurrently in the human and chimpanzee lineages independently. To distinguish between these two scenarios, we sequenced the fused sequences in the deleted haplotypes from multiple human and chimpanzee samples to identify the breakpoints of the chimpanzee and human deletions. If indeed these deletions are identical by descent, they will have exactly the same breakpoints. In other words, the likelihood of recurrent deletions to have exactly the same breakpoints is very small [27]. We sequenced the breakpoint junctions of the deleted haplotypes in both humans and chimpanzees (see Methods for details). The resulting deleted sequence is essentially a combination of two highly similar segmental duplications flanking the deletion. Consequently, even though we were able to produce the sequence, we could not identify the exact location of the breakpoint at the base pair scale given that the sequences of the segmental duplicates are near-identical.

**Fig. 2:**
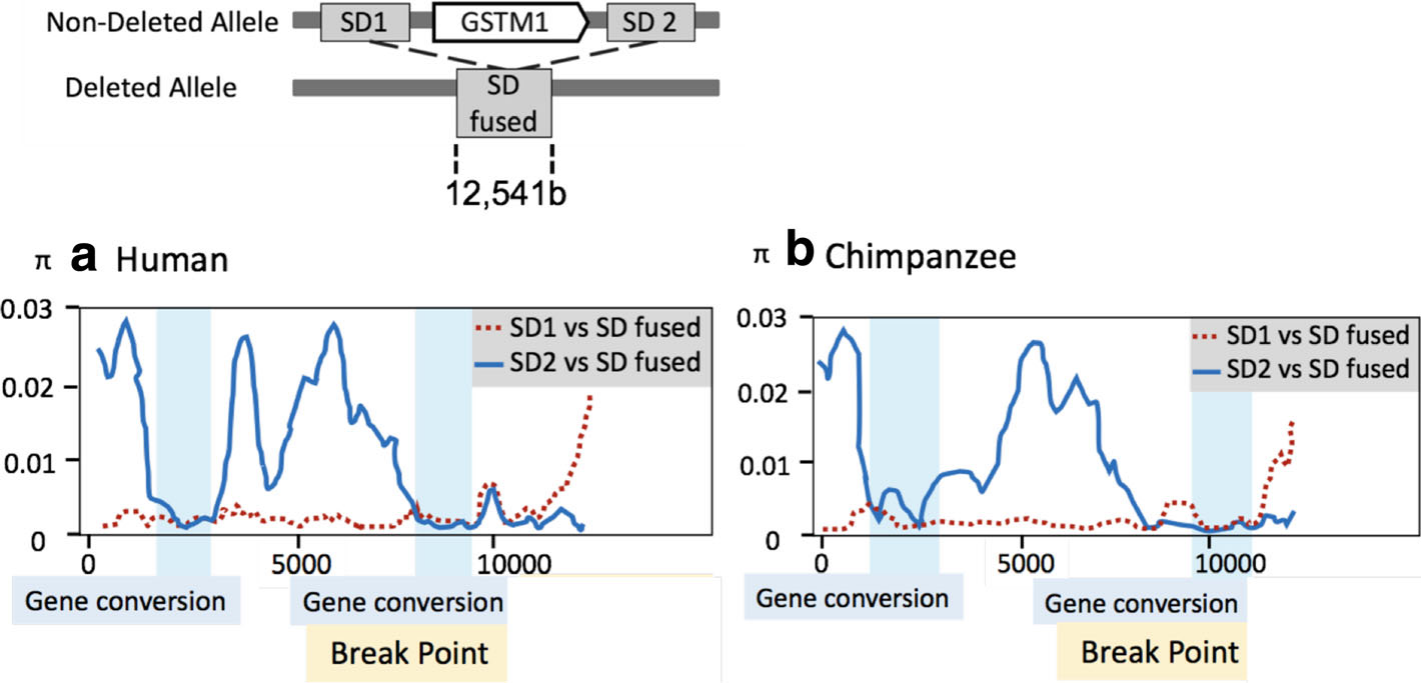
Sliding window analysis of nucleotide diversity (*π*) of (**a**) humans and (**b**) chimpanzees. The upper panel shows a schematic view of the compared sequences. This schema is accurate for both human and chimpanzee comparisons. Nucleotide diversity (*π*) between aligned 12,541b segmental duplications (window length: 500b, step size: 100b) is described. X-axis indicates chromosomal location and Y-axis indicates *π* values. The red dashed line indicates nucleotide diversity between segmental duplication (SD)1 and fused SD. The blue solid line indicates nucleotide diversity between human SD2 and fused SD. The putative gene conversion regions and breakpoint of the SD1 and SD2 fusion were marked in blue and yellow respectively. As indicated by blue-boxes, two gene conversion decrease the local similarity of SD1 and SDs, and the deleted allele was generated by the fusion of SD1 and SD2 at the breakpoint highlighted in yellow in both humans and chimpanzees

To narrow down the breakpoints, we examined the nucleotide differences between flanking segmental duplications (SD1 and SD2, defined as the SDs on upstream and downstream of the *GSTM1* gene in this paper) with each other and also with the fused sequence in the deleted haplotypes (fused SDs). We conducted this analysis for both humans and chimpanzees by means of sliding windows of 500 bp (Fig. 2). We found that there were islands of high sequence similarity in the windows at the ends of the segmental duplications when compared to each other in both human and chimpanzee genomes (Fig. 2). We argue that this observation is best explained by a gene conversion event between these two segmental duplications in the human-chimpanzee ancestor.

Identifying highly homologous sections of the fused SDs between a segmental duplication on one side of the *GSTM1* and the other would allowed us to infer the breakpoint of the deletion. More specifically, a segment of the fused segmental duplication should have more sequence similarity to the corresponding ancestral segmental duplication. Moving along the fused segmental duplication in a systematic fashion, we were able to find a region where the similarity pattern switches. Using this approach, we were able to narrow down the breakpoint where the two ancestral segmental duplication fused in both chimpanzee and human chromosomes independently (Fig. 2). This region is coincident with the previously reported putative breakpoint in European individuals [6]. However, this analysis still could not resolve the exact breakpoints of the deletion, and as such, our attempts to distinguish between identity by descent and recurrence scenarios using breakpoint sequences were not conclusive.

### Phylogenetic analyses suggested independent deletion formations of the *GSTM1* in humans and chimpanzees

**Fig. 3:**
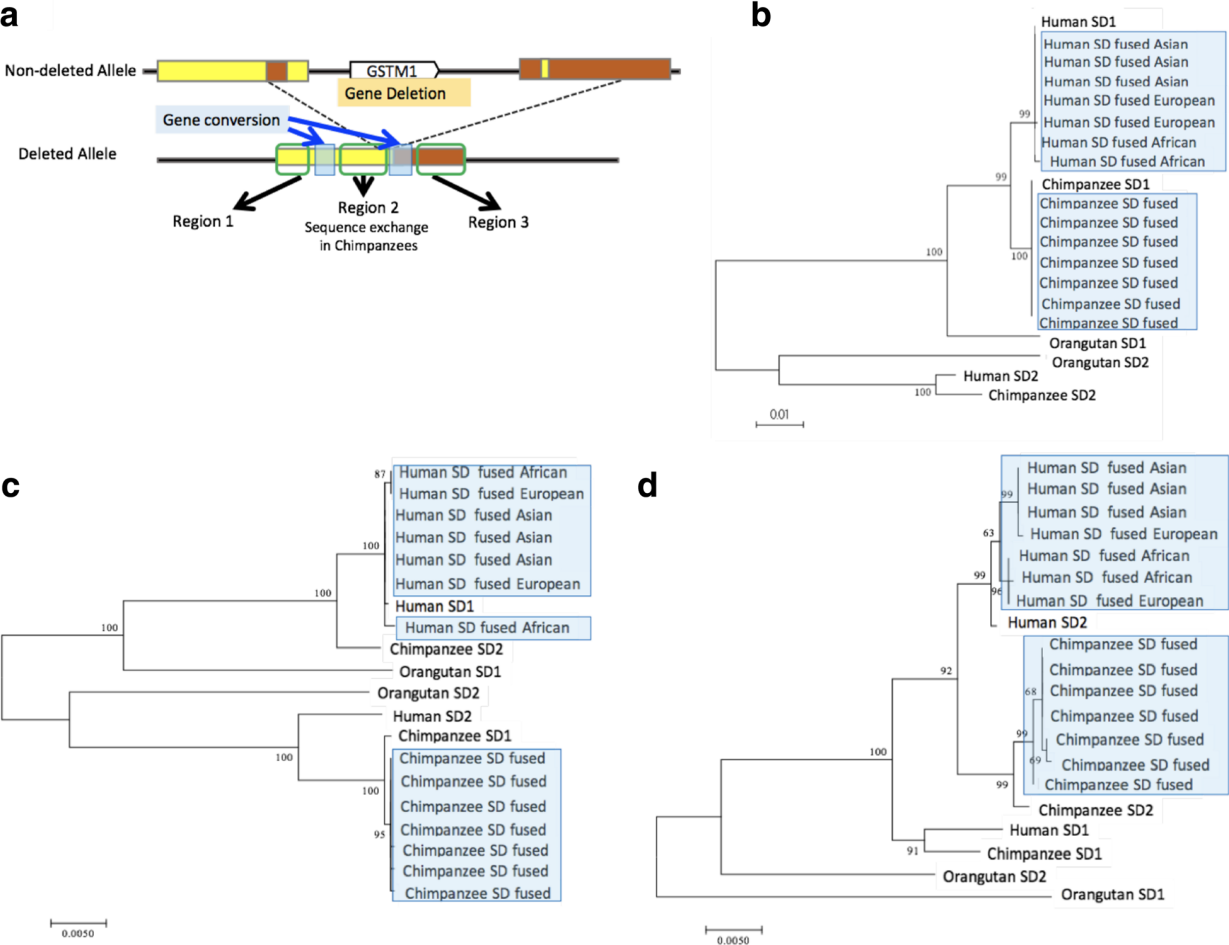
Maximum likelihood trees of the human and chimpanzee segmental duplications. **a** We divided the segmental duplication(s) into three regions: region 1 (0-1000b), region 2 (4001-7500b), and region 3 (12000b-) based on the boundaries we predicted. Then we reconstructed maximum-likelihood trees for (**b**) region 1, (**c**) region 2, and (**d**) region 3. We broke our analyse into these three regions to avoid any complications due to gene conversions, which we depicted by transparent light blue boxes. The input was human SD1 and SD2 from the reference genome, chimpanzee SD1 and SD2 from the reference genome, seven (three Asian, two European, two African) human fused SDs and seven chimpanzee fused SDs which we sequenced. Human and chimpanzee fused SDs are highlighted with blue color. Bootstrap values were described in the roots of the branches. These analyses support the recurrent segmental duplication fusion events (or in other words deletions) in human and chimpanzee lineages

To further investigate whether human and chimpanzee *GSTM1* deletion polymorphisms are identical-by-descent or recurrent, we employed a phylogenetic approach. As we described above, the deletion results in the fusion of two segmental duplications flanking the deleted region. We reasoned that if the deletions are identical by descent, the sequence resulting from the fusion of the segmental duplications from chimpanzees and humans should cluster together in a phylogenetic tree. To test this, we constructed phylogenetic trees of the fused segmental duplication sequences (fused SD) together with the segmental duplication on the 5′ of the *GSTM1* (SD1) and segmental duplication on the 3′ of the *GSTM1* (SD2). Taking the gene conversion detected in this locus (Fig. 2) into consideration, we divided the aligned region into three, region 1 (0-1000b), region 2 (4001-7500b), and region 3 (12000b-) and constructed trees for the regions separately (Fig. 3).

Trees constructed from both region 1 and 3 gave concordant results with very high bootstrap support (Fig. 3b and d). Specifically, all of the fused SD sequences from 7 humans that we sequenced clustered with human SD1 and SD2 sequences, while all of the fused SD sequences from 7 chimpanzees that we sequenced clustered with chimpanzee SD1 and SD2 sequences. These results contradict the identity-by-descent scenario, and instead support the scenario where the deletions were independently formed in human and chimpanzee lineages.

The alignments from region 2 supports strongly such independent recurrence scenario as well (Fig. 3c). However, for the region 2, we observed that the human SD1 is more similar to chimpanzee SD2 than it is to chimpanzee SD1, and that human SD2 is more similar to chimpanzee SD1 than it is to chimpanzee SD2, inconsistent with the results from the alignments from region 1 and 3 (Fig. 3c). A combination of multiple gene conversion events or sequence exchanges between paralogous duplicates may explain this observation. Such complex structural evolution through multiple gene conversion events has previously been described for Rh blood group locus in apes [43].

Using our alignments, we were able to estimate the age when the deletion variants were formed both in the chimpanzee and human lineages. To do this, we estimated the coalescent times of the fused SDs in each of the species. For this analysis, we used alignments from region 1 (Fig. 3b) that were used for the tree construction. We did not use region 2, because this region showed evidence for sequence exchange between segmental duplications, which may affect the age estimate. We did not use region 3, because it was relatively short, which reduces our power. To estimate the age, we first calculated the pairwise nucleotide differences between SD1 and fused SD haplotypes within each species. Based on the differences, we estimated the divergence time of the fused sequence using both the previously reported average mutation rate in primates [44] and also by the observed pairwise differences between chimpanzee and human SD1 haplotypes by MEGA7.0 [45]. The estimated ages of the fused SD were 364 k - 510 k and 341 k - 575 k years before present for humans and chimpanzees, respectively. We also estimated the divergence time of the fused sequence using the previously reported divergence time of humans and chimpanzees (6.3 million ago) [46]. With this method, the estimated ages of the fused SD were 343 k - 363 k and 360 k - 383 k years before present for humans and chimpanzees, respectively. More noteworthy, our results show that the coalescence times of the independent deletion events in humans and in chimpanzees overlap with each other. It is important to note here that the dates calculated here are prone to error as we do not know the exact mutation rate in this locus and that gene conversion events may affect the results.

### The sharing of *GSTM1* gene deletion among humans and chimpanzees is a rare occurrence

Next, we aimed to evaluate whether the deletion sharing between humans and chimpanzees that we observed for the *GSTM1* locus is unusual across the genome. It should be noted here that the similarity of the breakpoints of the chimpanzee and human *GSTM1* deletions was unexpected. In fact, we calculated that the probability of a recurrent breakpoint of an SV to co-occur in humans and chimpanzee lineages independently is less than 0.01 (see Methods). This is true, even when we do not consider that two (not only one) of the breakpoints of *GSTM1* deletion coincide in humans and chimpanzees. Moreover, the likelihood of this breakpoint sharing is even less given that the *GSTM1* deletion is polymorphic in both chimpanzees and humans.

Therefore, we wanted to know whether other polymorphic deletions with similar breakpoints are shared among humans and chimpanzees. To do this, we compared 1000 Genomes deletion polymorphism data [4] with polymorphic deletions reported for chimpanzees [39]. We chose a stringent, 70% reciprocal overlap threshold to account for the very similar breakpoint locations that we observed for human and chimpanzee *GSTM1* deletion. We found that only 12 of the 1713 polymorphic chimpanzee deletions overlap with 42,441 human deletions (Additional file 1: Figure S4). Based on this empirical observation, we conclude that less than 1% of the polymorphic deletions in chimpanzees is expected to be also polymorphic with similar breakpoints in humans. The *GSTM1* was one of the 12 shared deletions, confirming our PCR-based results. Moreover, when we further subset our dataset to account for only genic deletions that are common in human populations, we are left with only two deletions overlapping with the *GSTM1* and *UGT2B17.* This is noteworthy, particularly because the latter gene is surprisingly a member of another metabolizing gene family with similar functional attributes to *GSTM1.* We believe that the non-neutral forces (if any) that have maintained these polymorphic deletions remain a highly important next venue of research.

## Discussion

Here, we scrutinized the evolution of the *GSTM1* locus, including multiple gene conversion and structural variation events. By doing so, our work sheds light on the evolutionary diversification of a metabolizing *GSTM* gene family.

It is important to make a side note with regards to alignments because multiple analyses we used depend on the accuracy of these alignments. As mentioned earlier, the sequences of both segmental duplications and the fused-segmental duplications observed in the deleted chromosomes are very similar to each other (Additional file 2). However, a manual curation of the alignments was necessary due to the small (10–20 bp) insertions and deletions between paralogues and orthologues as exemplified in Additional file 1: Figure S5. The presence and absence of these insertions and deletions in different sequences match well with the results of single nucleotide variant based analyses (e.g., the insertions and deletions fit well with the single nucleotide-based phylogenetic trees, data not shown).

**Fig. 4:**
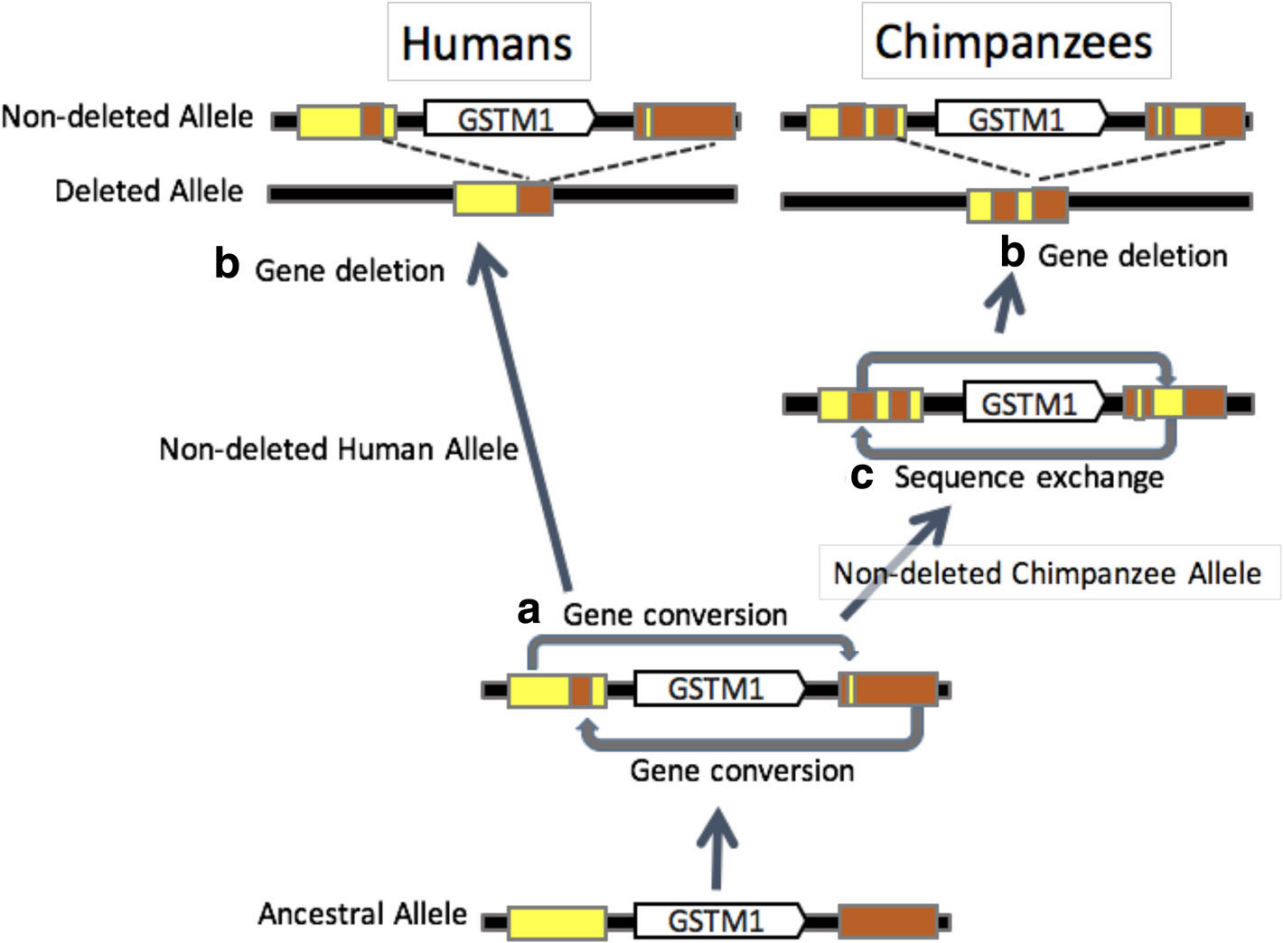
An evolutionary model of the *GSTM1* deletion in humans and chimpanzees. Based on our results, we built a model to describe the mutational events through evolutionary time: (**a**) Gene conversions occurred between SD1 and SD2 before the divergence of humans and chimpanzees. (**b**) The *GSTM1* gene was deleted in both human and chimpanzee lineages with very similar breakpoints. (**c**) A chimpanzee-specific sequence exchange between the SD1 and SD2 occurred

Considering all our results summarized in Figs. 1a, 2a,b and 3a-d, we were able to build a model for the evolution of this locus since the human-chimpanzee ancestor (Fig. 4). Our model assumes an ancestral state of the locus where there is an intact *GSTM1* gene, which was flanked with two ancestral SDs (Fig. 1a). Based on our analyses, we concluded that there were two gene conversion events between these segmental duplications before the human and chimpanzee speciation. We based this conclusion on the observation that there are two subregions of SD1 and SD2 that are much similar to each other as compared to rest of the sequence in these duplicated segments (Fig. 2a and b). Following this, independent *GSTM1* gene deletion events generated the current polymorphisms in both species (Fig. 3). In the chimpanzee lineage, we argue that an additional sequence exchange between SD1 and SD2 happened before the deletion event. We based this on the observation in the phylogenetic trees that for region 1 and region 3, chimpanzee SD1 has a similar sequence to human SD1 (Fig. 3b and d), but chimpanzee SD1 clusters with human SD2 for the region 2 (Fig. 3c). This observation is the best explained by a sequence exchange affecting region 2 in the chimpanzee lineage.

If these two deletions have happened in humans and chimpanzees independently after the species divergence, the lower bound on the mutation rate for the deletion can be calculated as 2/12.6 myr = 1.58 × 10^− 7^ mutations per year. This rate does not take into account potential mutations that occurred in chimpanzee and human lineages, but got lost in the contemporary populations. It was not considered that the both the human and chimpanzee deletions we observe occurred much later than the human chimpanzee divergence, which we used to calibrate our calculations. Both of these exclusions lead us to calculate a very conservative (*i.e.*, slower) mutation rate. Even then, this rate is more than 100 times higher than the average nucleotide substitution rate [44] and at least 1000 times higher than the mutation rate estimated for structural variants (> 500 bp) in the human genome [47]. Overall, our results contribute to the observation that the deletion rate in this locus is high, which is concordant with our cross-species analyses where we report multiple large structural events shaping the architecture of the *GSTM* locus in primates.

## Conclusion

The *GSTM1* locus that we describe in this study harbors one of the few common gene deletions found in the human genome [48]. Our finding that this gene is also deleted in chimpanzees is noteworthy. In fact, our genome-wide analysis indicates that this is an unusual case, where we found only two such polymorphic deletions shared in human and chimpanzee populations, one of them overlapping *GSTM1* and the other overlapping another metabolizing gene, the *UGTB17.* Indeed, structural variants observed in metabolizing gene families, including the *UGT* [49, 50], the *CYP* [51] and the *NAT* [25], contribute significantly to the functional diversity of these gene families and have been discussed within an non-neutral framework.

Combining these insights, we argue that our findings can be generalizable to gene families that are comprised of similar, tandemly-located genes with environmental interaction functions. There is accumulating evidence that almost all such gene families have been rapidly evolving through complex structural variations [52], creating lineage-specific repertoires of functional diversity [35, 53]. Further studies, perhaps involving population level long-read sequencing in multiple species, are needed to gain a better understanding of the evolutionary feature of tandem genes with environment-interaction functions, and eventually their evolutionary impact.

## Methods

### Sample information

A total of 37 unrelated chimpanzees (36 *Pan troglodytes verus* and a *Pan Troglodytes troglodytes*, and seven human samples (three Southeast Asian individuals, two European individuals, and two African individuals)) were used in this study for the sequencing. The human samples were collected after informed consent was obtained. DNA samples used for sequencing are stored in the Primate Cell & DNA Repository of Unit of Human Biology & Genetics, Department of Biological Sciences, Graduate School of Science, The University of Tokyo. Four Chimpanzee DNA for ddPCR and Read-depth method were obtained from Coriell (PR00226, PR00738, PR00818 and PR1171). Detailed information of the sample was described in [39].

### PCR amplifications

A multiplex PCR was conducted to determine the presence of the *GSTM1* wild-type alleles. β-globin gene was used as an internal control and was amplified simultaneously; Only samples with β-globin PCR positive results were included in the following analysis. PCR primers are shown in Additional file 1: Table S3 [37, 38]. We followed the standard PCR protocol and gel electrophoresis.

### ddPCR amplifications

To genotype the *GSTM1* deletion in four chimpanzees, we used ddPCR with the following primers for the *GSTM1*: forward TCGAGGGTGCCATTACATTC and reverse ACTTCTGTCCTGGGTCATTC. We followed standard protocol provided by Bio-Rad EIF2C1 probe assay.

### Read-depth methods

To genotype the *GSTM1* deletion in four chimpanzees, we used the BAM files created by [39]. We calculated averaged read-depth within the *GSTM1* gene (chr1:127,159,466–127,164,895 in PanTro3) and that of the entire genome and divided the depth in the *GSTM1* by the depth of the entire genome.

### DNA sequencing

Fused segmental duplications of seven heterozygous chimpanzees and humans (three Southeast Asian individuals, two European individuals, and two African individuals) were sequenced by primer walking method (Primers are described in Additional file 1: Table S3). The sequences were uploaded to DDBJ (http://www.ddbj.nig.ac.jp/). Sequencing analyses were conducted by Eurofin Genomics. The sequences generated during the current study are available in the DDBJ (http://www.ddbj.nig.ac.jp/) and the accession numbers are LC312398-LC312411).

### Detection of the human-chimpanzee shared deletion polymorphisms

To evaluate the rarity of deletion polymorphisms shared between humans and chimpanzees, we took deletion polymorphism data of chimpanzees from a previous paper [39], then lifted these deletions to the human reference genome (hg19) by LiftOver. The data for this paper can be found in Primate Structural Variation Database (http://www.korbel.embl.de/primate_sv/). Of the 2770 chimpanzee polymorphic deletions, we were able to lift-over 1713 deletions. We compared the 1713 deletion from chimpanzee data and human deletions reported in phase3 1000 genomes (42,441 deletions) [4] with 70% reciprocal match.

### Comparative analysis of the primate *GSTM* homologous genes

To obtain the *GSTM* gene sequences in primates, we modified a pipeline described earlier by [54]. Specifically, the human *GSTM1* coding gene sequence was used as a reference and as input for tblastn [55] searches against eight primate genomes and a tree shrew genome available in GenomeNet (http://www.genome.jp/). The genomes that are used are as follows: *Homo sapiens* [56], *Pan troglodytes* [57], *Pan paniscus* [58], *Gorilla gorilla gorilla* [59], *Pongo abelii* [60], *Nomascus leucogenys* [61] *Macaca mulatta* [62], *Callithrix jacchus* [63] and *Tupaia belangeri chinensis* [64]). The tblastn results were used as inputs for blastx against the genome sequence of *H. sapiens*. This allows us to verify that these input primate sequences are indeed members of the *GSTM* family. In addition, the orthologs of different *GSTM* genes in nonhuman primates were identified based on the blastx results. If the top blastx hit was not a human *GSTM* sequence, the sequence was excluded. In the subsequent analysis, we considered only sequences that contain both GST N-domain and GST C-domain. We also annotate pseudogenes by documenting sequences with premature stop codons and gene truncations relative to the functional *GSTM* gene (Additional file 1: Table S2). Chromosomal locations of each of the *GSTM* genes found in primates could be obtained (except for bonobo due to reference genome quality) using NCBI database (http://www.ncbi.nlm.nih.gov/gene).

A similar approach was used in the detection of the segmental duplications. Briefly, the 2 kb highly similar segmental duplications in humans which were reported in [6] were used as inputs for blast search against the primate genomes to detect segmental duplications in nonhuman primates. Phylogenetic trees were constructed (Additional file 1: Figure S1) using the ML and NJ methods in MEGA7 [45].

### Comparative analysis of the human and chimpanzee segmental duplications

The entire sequence of a *H. sapiens* fused segmental duplication was used as input for blast search against primate genomes. Segmental duplication sequences in the reference genomes of *H. sapiens, Pan troglodytes* and *Pongo abelii* were conserved enough to align to each other. Nucleotide sequences of 7 human fused segmental duplications, seven chimpanzee fused segmental duplications that we sequenced along with the human, chimpanzee, orangutan segmental duplications retrieved from the reference genomes were aligned manually in MEGA7 [45]. Some indels (10-20 bp) in the SDs needed manual alignments (Additional file 1: Figure S4). Specifically, ClastalW [65] in the MEGA7 software [45] could not align these sequences directly, even when we allowed indels. Instead, by manually inserting gaps for the indels produced highly consistent alignments as we gave examples in Additional file 1: Figure S4. The pattern of indels of each sequence fits with the phylogenetic relationship of the same region constructed without indel information (Fig. 3b-d). To ensure the accuracy of our results, we also conducted an alignment with MAFFT [66] and reproduced the phylogenetic analyses in Fig. 3 with MAFFT alignments as well (Additional file 1: Figure S6). The results are consistent between different alignment methods. Sliding window analyses of pairwise differences of the sequences (simple count) were conducted for manually aligned segmental duplications in order to calculate nucleotide diversity between segmental duplication by DnaSP [67].

Based on the results of sliding window analysis, we divided the whole 12,541b segmental duplications into three parts (region 1: -1000b, fused SD comes from SD1 in humans, region 2: 4001b-7500b, fused SD comes from SD1 in humans and lineage-specific sequence exchange occurred in human or chimpanzee lineage, region 3: 10000b- fused SD comes from SD2 in humans). We conducted phylogenetic analyses of the sequences of the segmental duplications using ML methods in MEGA7 with the following models: 100 bootstrap replications, general time reversible model of nucleotide substitution, gamma distribution of rates, partial deletion with cutoff 95%, Nearest-Neighbor-Interchange, Make initial tree automatically [45].

### Estimation of the probability of observing deletions with a similar upstream breakpoint recurrently evolved in chimpanzees and humans

We estimated the chances of a deletion with a similar upstream breakpoint to recurrently evolve independently in chimpanzees and humans given a particular mutation rate. Kloosterman et al. [47] estimated a mutation rate of 0.041 mutations per haploid genome per generations for structural variants that are larger than 500 bp. As such, we surmised that the mutation rate for the upstream breakpoints for these SVs will be identical. Based on this, we assumed a series of mutation rates ranging from 0.001 to 0.2. We estimated that the breakpoints of the *GSTM1* deletion is similar in chimpanzees and humans with about 4000 base pairs resolution (Fig. 2). Based on these conservative assumptions and assuming a generation time of 20 (for both chimpanzees and humans) and divergence time of 7 million years between these two species, we calculated the probability of breakpoints to reoccur with the following formula:

$$ {\displaystyle \begin{array}{l}\Big\{\left(\left(\mathrm{mutation}\ {\mathrm{rate}}^{\ast}\mathrm{diploid}\ \mathrm{genome}\right)/\mathrm{generation}\ \mathrm{time}\right)/\mathrm{size}\ \mathrm{of}\ \mathrm{the}\ {\mathrm{genome}}^{\ast}\mathrm{breakpoint}\ \mathrm{noise}\\ {}\left(4,000\mathrm{bp}\right)/7\ \mathrm{million}\ \mathrm{years}\Big\}\hat{\mkern6mu} 2\end{array}} $$

Based on this, we have a function of probability based on the mutation rate as portrayed by the figure below. It indicates that having a single deletion to occur recurrently with shared breakpoints is unlikely (Additional file 1: Figure S7). And this is a very conservative estimate, given that (i) we are not considering the other breakpoint is also similar and (ii) we are not incorporating the fact that this variation remains polymorphic in both species.

## Additional files

Additional file 1: **Table S1.** The functional GSTM analyzed. **Table S2.** The GSTM pseudogenes detected. **Table S3.** PCR primers and sequencing primers. **Figure S1.** NJ and ML tree of the primate and tree shrew *GSTM* genes. **Figure S2.** A dotplot of Humans and Chimpanzees *GSTM* gene cluster. **Figure S3.** Genotyping results in chimpanzees by ddPCR and read-depth approaches. **Figure S4.** The length and frequency of the deletions shared between humans and chimpanzees. **Figure S5.** The manual alignment of the SDs of humans, chimpanzees and orangutans. **Figure S6.** Maximum likelihood trees of the human and chimpanzee segmental duplications using MAFFT [66]. **Figure S7.** The probability of a deletion with a similar upstream breakpoint to recurrently evolve independently in chimpanzees and humans given a particular mutation rate. (ZIP 2977 kb)

Additional file 2: Sequence alignment of the GSTM1 region of 7 deleted humans and 7 deleted chimpanzees and reference genomes of both species. (TXT 252 kb)

## Abbreviations

CYP: Cytochrome P
ddPCR: Droplet Digital PCR
GSTM1: Glutathione S-transferase Mu 1
GSTT1: Glutathione S-transferase Theta 1
NAT: N-acetyltransferase 2
SD: Segmental duplication
UGT: Uridine diphosphoglucuronosyltransferase
