# Supplementary material for: Complex evolution of the GSTM gene family involves sharing of GSTM1 deletion polymorphism in humans and chimpanzees
Source: BMC Genomics. 2018 Apr 25;19:293. doi: 10.1186/s12864-018-4676-z (PMC5918908; doi:10.1186/s12864-018-4676-z)
Supplement: Supplementary file 1 — Table S1. The functional GSTM analyzed. Table S2. The GSTM pseudogenes detected. Table S3. PCR primers and sequencing primers. Figure S1. NJ and ML tree of the primate and tree shrew GSTM genes. Figure S2. A dotplot of Humans and Chimpanzees GSTM gene cluster. Figure S3. Genotyping results in chimpanzees by ddPCR and read-depth approaches. Figure S4. The length and frequency of the deletions shared between humans and chimpanzees. Figure S5. The manual alignment of the SDs of humans, chimpanzees and orangutans. Figure S6. Maximum likelihood trees of the human and chimpanzee segmental duplications using MAFFT [66]. Figure S7. The probability of a deletion with a similar upstream breakpoint to recurrently evolve independently in chimpanzees and humans given a particular mutation rate. (ZIP 2977 kb) [file 12864_2018_4676_MOESM1_ESM.zip › STables.docx]

**Table S1. The functional *GSTM* analyzed.**

| Species | Gene ID | Ortholog | Exon | Chromosome |
| --- | --- | --- | --- | --- |
| *Homo sapiens* | hsa_2944 | GSTM1 | 8 | 1 |
| *Homo sapiens* | hsa_2946 | GSTM2 | 10 | 1 |
| *Homo sapiens* | hsa_2947 | GSTM3 | 9 | 1 |
| *Homo sapiens* | hsa_2948 | GSTM4 | 9 | 1 |
| *Homo sapiens* | hsa_2949 | GSTM5 | 9 | 1 |
| *Pan troglodytes* | ptr_745723 | GSTM1 | 9 | 1 |
| *Pan troglodytes* | ptr_457128 | GSTM3 | 8 | 1 |
| *Pan troglodytes* | ptr_457094 | GSTM4 | 9 | 1 |
| *Pan troglodytes* | ptr_745685 | GSTM5 | 9 | 1 |
| *Pan troglodytes* | ptr_100612597 | GSTM2 | 5 | 1 |
| *Pan paniscus* | pps_100992725 | GSTM1 | 8 | Not reported |
| *Pan paniscus* | pps_100969075 | GSTM2 | 1 | Not reported |
| *Pan paniscus* | pps_100987265 | GSTM2 | 5 | Not reported |
| *Pan paniscus* | pps_100992371 | GSTM3 | 9 | Not reported |
| *Pan paniscus* | pps_100967236 | GSTM4 | 9 | Not reported |
| *Pan paniscus* | pps_100993315 | GSTM5 | 9 | Not reported |
| *Gorilla* | ggo_101129341 | GSTM1 | 9 | 1 |
| *Gorilla* | ggo_101127551 | GSTM2 | 9 | 1 |
| *Gorilla* | ggo_101128753 | GSTM3 | 9 | 1 |
| *Gorilla* | ggo_101130552 | GSTM5 | 9 | 1 |
| *Pongo abelii* | pon_100453622 | GSTM1 | 8 | 1 |
| *Pongo abelii* | pon_100174562 | GSTM2 | 7 | 1 |
| *Pongo abelii* | pon_100452892 | GSTM3 | 8 | 1 |
| *Pongo abelii* | pon_100454483 | GSTM4 | 9 | 1 |
| *Pongo abelii* | pon_100453263 | GSTM5 | 8 | 1 |
| *Nomascus leucogenys* | nle_100587851 | GSTM1 | 16 | 12 |
| *Nomascus leucogenys* | nle_100587060 | GSTM2 | 8 | 12 |
| *Nomascus leucogenys* | nle_100588847 | GSTM3 | 9 | 12 |
| *Nomascus leucogenys* | nle_100586279 | GSTM4 | 10 | 12 |
| *Macaca mulatta* | mcc_700053 | GSTM2 | 8 | 1 |
| *Macaca mulatta* | mcc_700303 | GSTM3 | 8 | 1 |
| *Macaca mulatta* | mcc_699923 | GSTM4 | 9 | 1 |
| *Macaca mulatta* | mcc_100426406 | GSTM5 | 9 | 1 |
| *Callithrix jacchus* | cjc_100385358 | GSTM2 | 7 | 7 |
| *Callithrix jacchus* | cjc_100407453 | GSTM3 | 7 | 8 |
| *Callithrix jacchus* | cjc_100407094 | GSTM4 | 7 | 8 |
| *Callithrix jacchus* | cjc_100406483 | GSTM1 | 7 | 8 |
| *T. belangeri chinensis* | tup_102481112 | GSTM3 | 16 | Not reported |
| *T. belangeri chinensis* | tup_102479291 | GSTM3? | 16 | Not reported |

Abbreviations: hsa: human, ptr: chimpanzee, pps: bonobo, ggo: western lowland gorilla, pon: Sumatran orangutan, nle: northern white-cheeked gibbon, mmu, rhesus monkey, cjc: white-tufted-ear marmoset, tup tree shrew.

**Table S2. The *GSTM* pseudogenes detected.**

| **Species** | **Gene ID** | **Chromosome** |
| --- | --- | --- |
| *Homo sapiens* | hsa:442245 | 6 |
| *Pan troglodytes* | ptr:107971337 | 1 |
| *Pan troglodytes* | ptr:107973014 | Not reported |
| *Pan troglodytes* | ptr:107973268 | Not reported |
| *Pan troglodytes* | ptr:472094 | 6 |
| *Pan troglodytes* | ptr:744182 | 1 |
| *Pan paniscus* | pps:100971356 | 1 |
| *Gorilla gorilla gorilla* | ggo:101128395 | 1 |
| *Nomascus leucogenys* | nle:100580720 | 21 |
| *Nomascus leucogenys* | nle:100602332 | 3 |
| *Macaca mulatta* | mcc:100426893 | 2 |
| *Macaca mulatta* | mcc:106996104 | Not reported |
| *Macaca mulatta* | mcc:700178 | 1 |
| *Callithrix jacchus* | cjc:100393909 | 8 |
| *Callithrix jacchus* | cjc:100411587 | 14 |
| *Callithrix jacchus* | cjc:100895885 | 5 |
| *T. belangeri chinensis* | tup:102483043 | Not reported |
| *T. belangeri chinensis* | tup:102492543 | Not reported |
| *T. belangeri chinensis* | tup:106733134 | Not reported |

**Table S3. PCR primers and sequencing primers**

| primers | sequence 5'-3' |
| --- | --- |
| PCR forward (Roodi et al., 2004) | CCTGTTGAAGGAGCTTATGCTGAA |
| PCR reverse humans (Roodi et al., 2004) | TTCTGAGGACTGGACTGATGATC |
| PCR reverse chimpanzees (modified from Roodi et al., 2004) | TTCTGAGGACTGGACCGATGA |
| Sequencing primers for humans |  |
| r2 | CCATTTATAGACCTCCCCCC |
| r3 | ACTGGCTGGAACGGGACCT |
| r4 | GAAGTAGTGCCTGCTGTTG |
| r5 | GAAACAATACTGACAGACAAAA |
| r6 | TTGCAGTGAGCCGAGATAGT |
| r7 | GAGCTTGCTGGGGGAGAC |
| r8 | ACAACAAAGAATTCTGCCCTCC |
| r9 | AAGTCAATATATAATAAGCACC |
| r10 | TGGGGGTGGGAGGCGATG |
| r11 | AACAAAAAAGGTGAAGGTAGAA |
| r12 | CAAAGTCACCAAAAACGGGAAA |
| r13 | AATCTAAGAGTAAACACGCACA |
| r14 | ACAGTGGACAGGCCTTAAGT |
| r15 | GTCGGTCCTTTCAATGTCAG |
| r16 | AAACAAAAATACAAAAATCGGCT |
| r17 | TTCCCCTGCACACGCCCTC |
| r18 | TCCTTTAGAACCCAGCCAC |
| r19 | GGCTGGACTTCATGTAGGC |
| r20 | GGAGGCTCTTGGCTTGTC |
| r21 | CTTGACCCTGGCACATAAC |
| r22 | CATCTCACAGGGGGAACAC |
| 9-10 | GATGGCTGGTAAATTATTGTG |
| 11-12 | CTACTTCTAATATTGATGGCTA |
| 12-13 | CTCTTCTTGTTCACTGATGTA |
| 12-13to12 | CTCCGGGCCTCAAGTTATC |
| Sequencing primers for chimpanzees |  |
| PANr12 | CAAAGTAACCAAAAATGGGAAG |
| PANr16 | AAACAAAAATACAAAAATTAGCT |
| PANr18 | TCTTTTAGAACCCAGCCAC |
| PAN12-13to12 | ATCTGCCCACCTCTACCCCT |
