# Supplementary figures and images for: Complex evolution of the GSTM gene family involves sharing of GSTM1 deletion polymorphism in humans and chimpanzees
Source: BMC Genomics. 2018 Apr 25;19:293. doi: 10.1186/s12864-018-4676-z (PMC5918908; doi:10.1186/s12864-018-4676-z)

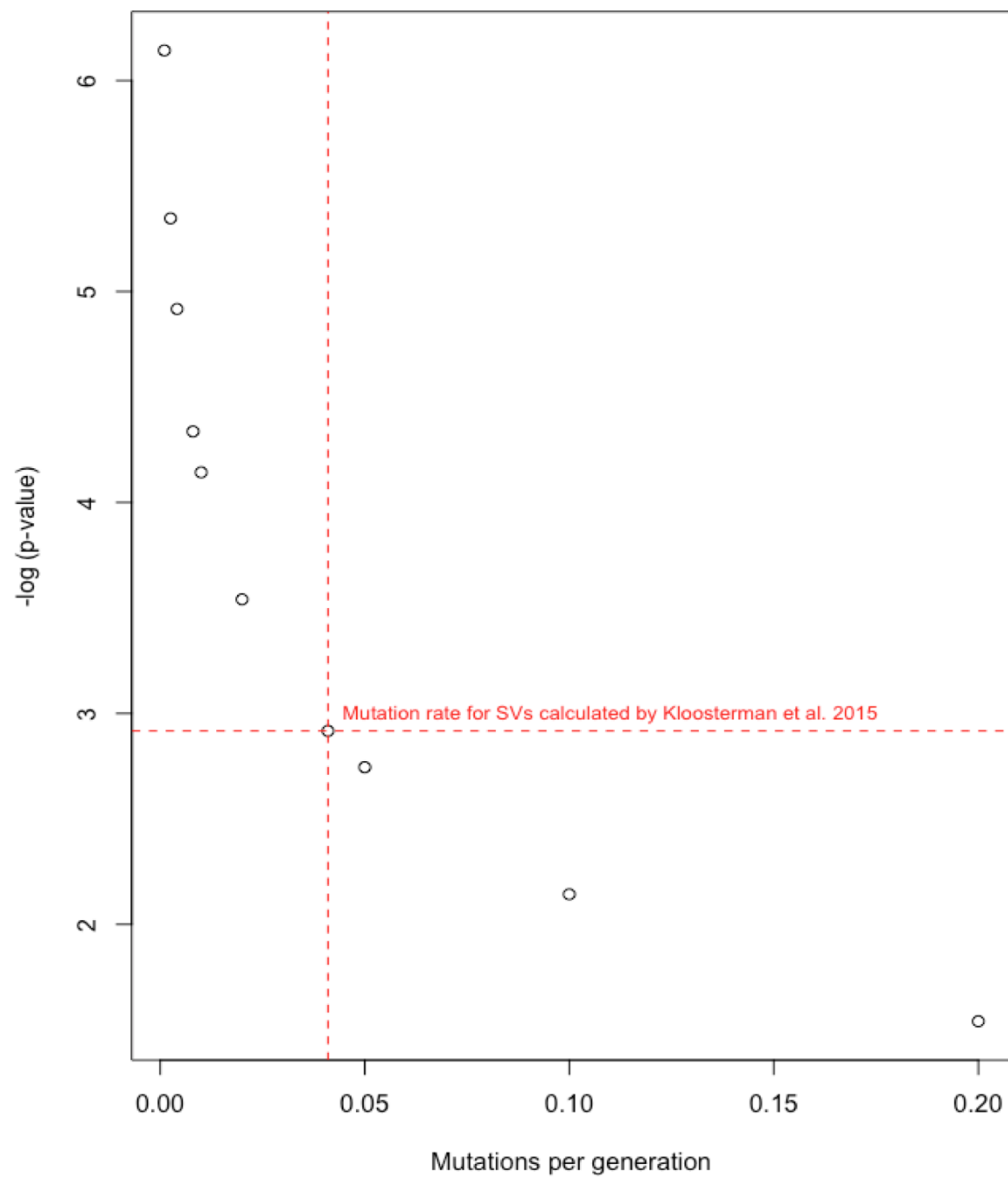

Supplement: Supplementary file 1 — Table S1. The functional GSTM analyzed. Table S2. The GSTM pseudogenes detected. Table S3. PCR primers and sequencing primers. Figure S1. NJ and ML tree of the primate and tree shrew GSTM genes. Figure S2. A dotplot of Humans and Chimpanzees GSTM gene cluster. Figure S3. Genotyping results in chimpanzees by ddPCR and read-depth approaches. Figure S4. The length and frequency of the deletions shared between humans and chimpanzees. Figure S5. The manual alignment of the SDs of humans, chimpanzees and orangutans. Figure S6. Maximum likelihood trees of the human and chimpanzee segmental duplications using MAFFT [66]. Figure S7. The probability of a deletion with a similar upstream breakpoint to recurrently evolve independently in chimpanzees and humans given a particular mutation rate. (ZIP 2977 kb) [file 12864_2018_4676_MOESM1_ESM.zip › Figure_S7.pdf]

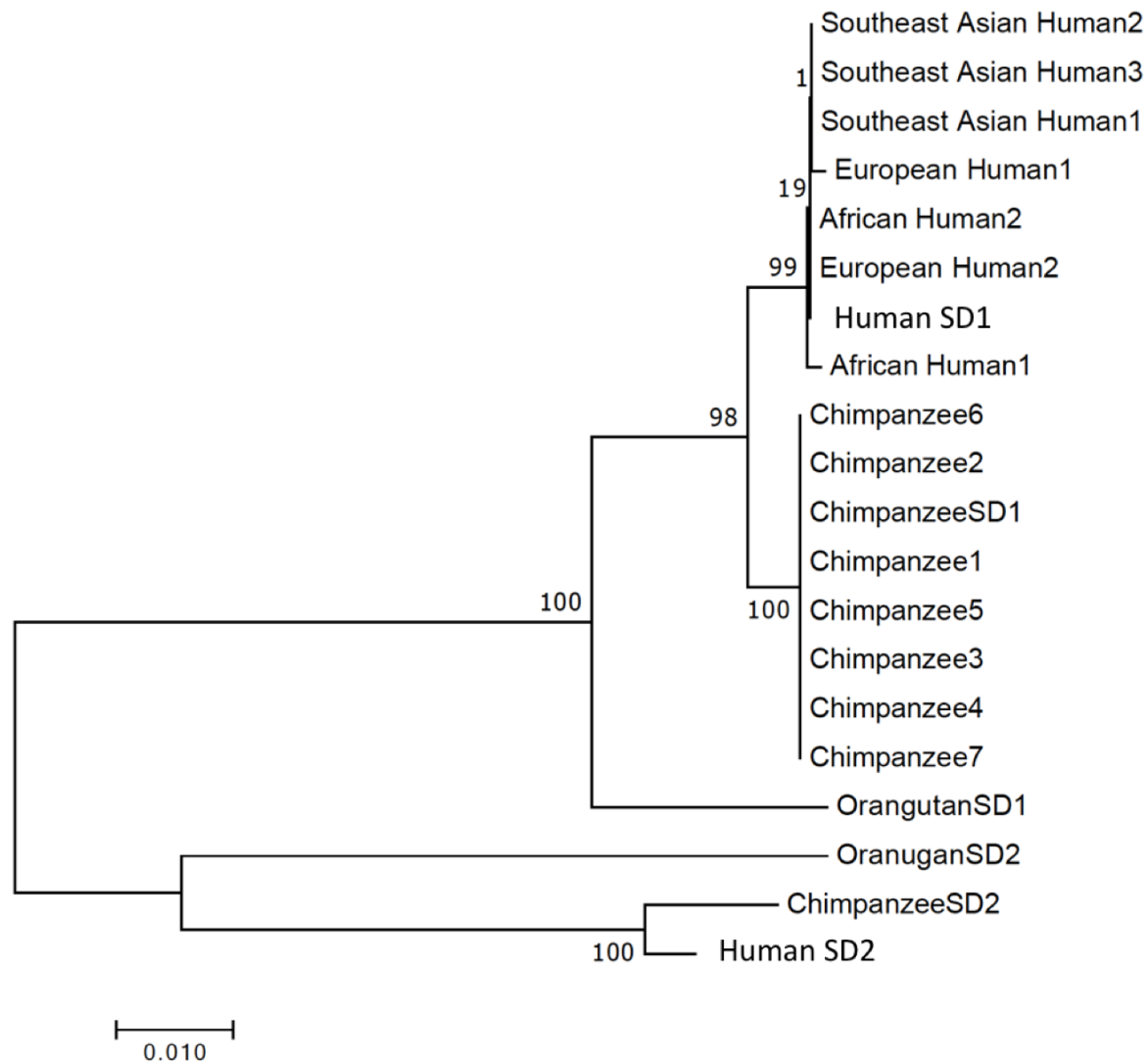

Supplement: Supplementary file 1 — Table S1. The functional GSTM analyzed. Table S2. The GSTM pseudogenes detected. Table S3. PCR primers and sequencing primers. Figure S1. NJ and ML tree of the primate and tree shrew GSTM genes. Figure S2. A dotplot of Humans and Chimpanzees GSTM gene cluster. Figure S3. Genotyping results in chimpanzees by ddPCR and read-depth approaches. Figure S4. The length and frequency of the deletions shared between humans and chimpanzees. Figure S5. The manual alignment of the SDs of humans, chimpanzees and orangutans. Figure S6. Maximum likelihood trees of the human and chimpanzee segmental duplications using MAFFT [66]. Figure S7. The probability of a deletion with a similar upstream breakpoint to recurrently evolve independently in chimpanzees and humans given a particular mutation rate. (ZIP 2977 kb) [file 12864_2018_4676_MOESM1_ESM.zip › Figure_S6.pdf]

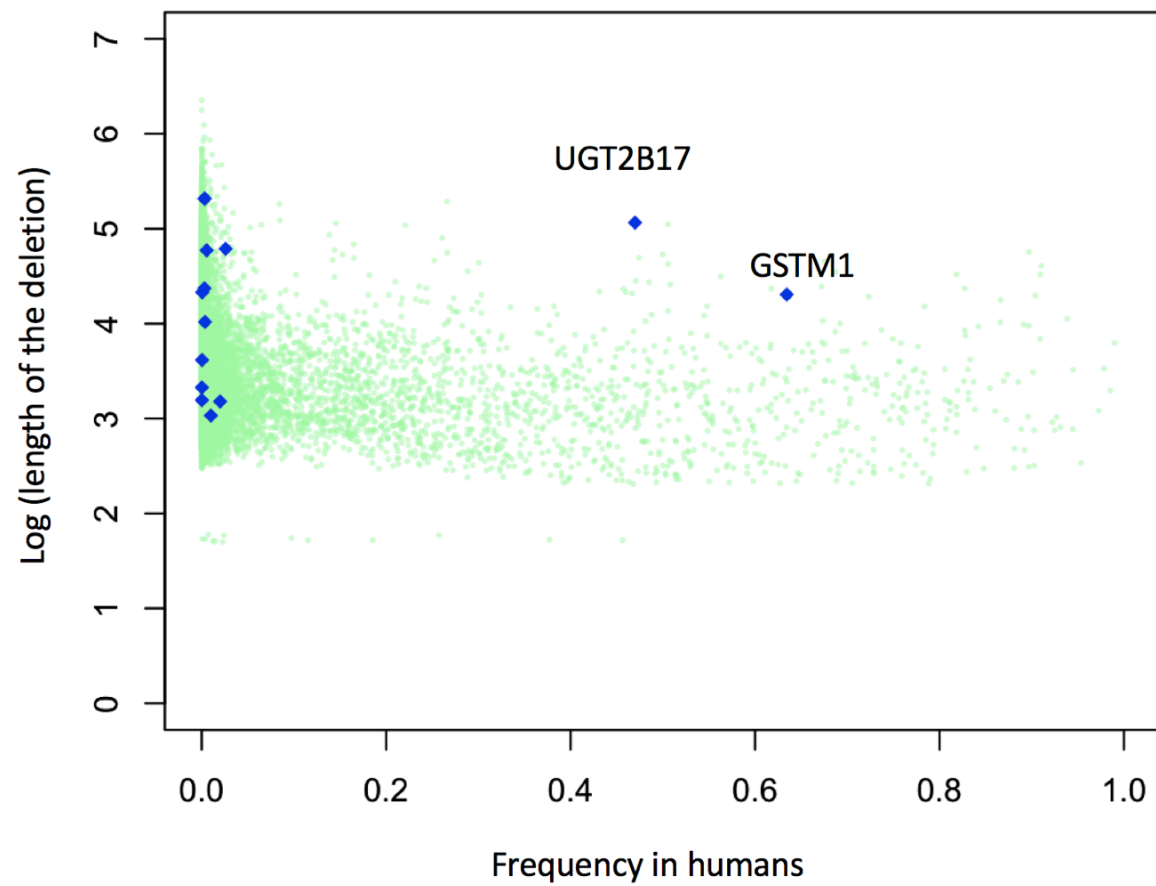

Supplement: Supplementary file 1 — Table S1. The functional GSTM analyzed. Table S2. The GSTM pseudogenes detected. Table S3. PCR primers and sequencing primers. Figure S1. NJ and ML tree of the primate and tree shrew GSTM genes. Figure S2. A dotplot of Humans and Chimpanzees GSTM gene cluster. Figure S3. Genotyping results in chimpanzees by ddPCR and read-depth approaches. Figure S4. The length and frequency of the deletions shared between humans and chimpanzees. Figure S5. The manual alignment of the SDs of humans, chimpanzees and orangutans. Figure S6. Maximum likelihood trees of the human and chimpanzee segmental duplications using MAFFT [66]. Figure S7. The probability of a deletion with a similar upstream breakpoint to recurrently evolve independently in chimpanzees and humans given a particular mutation rate. (ZIP 2977 kb) [file 12864_2018_4676_MOESM1_ESM.zip › Figure_S5.pdf]

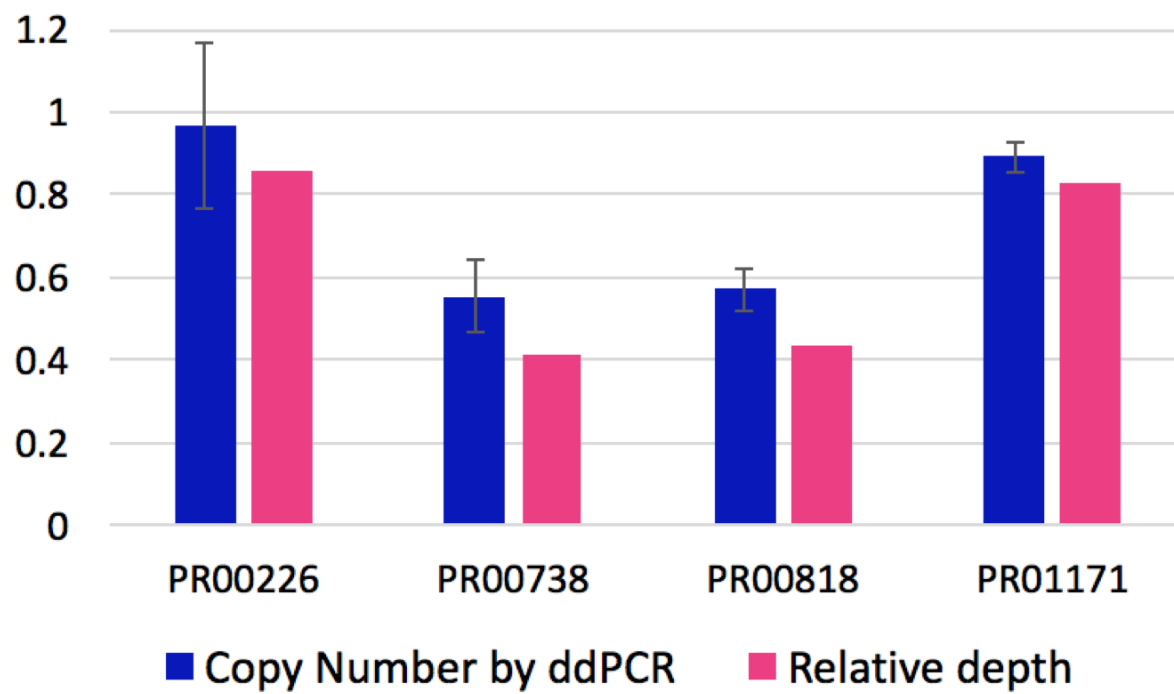

Supplement: Supplementary file 1 — Table S1. The functional GSTM analyzed. Table S2. The GSTM pseudogenes detected. Table S3. PCR primers and sequencing primers. Figure S1. NJ and ML tree of the primate and tree shrew GSTM genes. Figure S2. A dotplot of Humans and Chimpanzees GSTM gene cluster. Figure S3. Genotyping results in chimpanzees by ddPCR and read-depth approaches. Figure S4. The length and frequency of the deletions shared between humans and chimpanzees. Figure S5. The manual alignment of the SDs of humans, chimpanzees and orangutans. Figure S6. Maximum likelihood trees of the human and chimpanzee segmental duplications using MAFFT [66]. Figure S7. The probability of a deletion with a similar upstream breakpoint to recurrently evolve independently in chimpanzees and humans given a particular mutation rate. (ZIP 2977 kb) [file 12864_2018_4676_MOESM1_ESM.zip › Figure_S3.pdf]

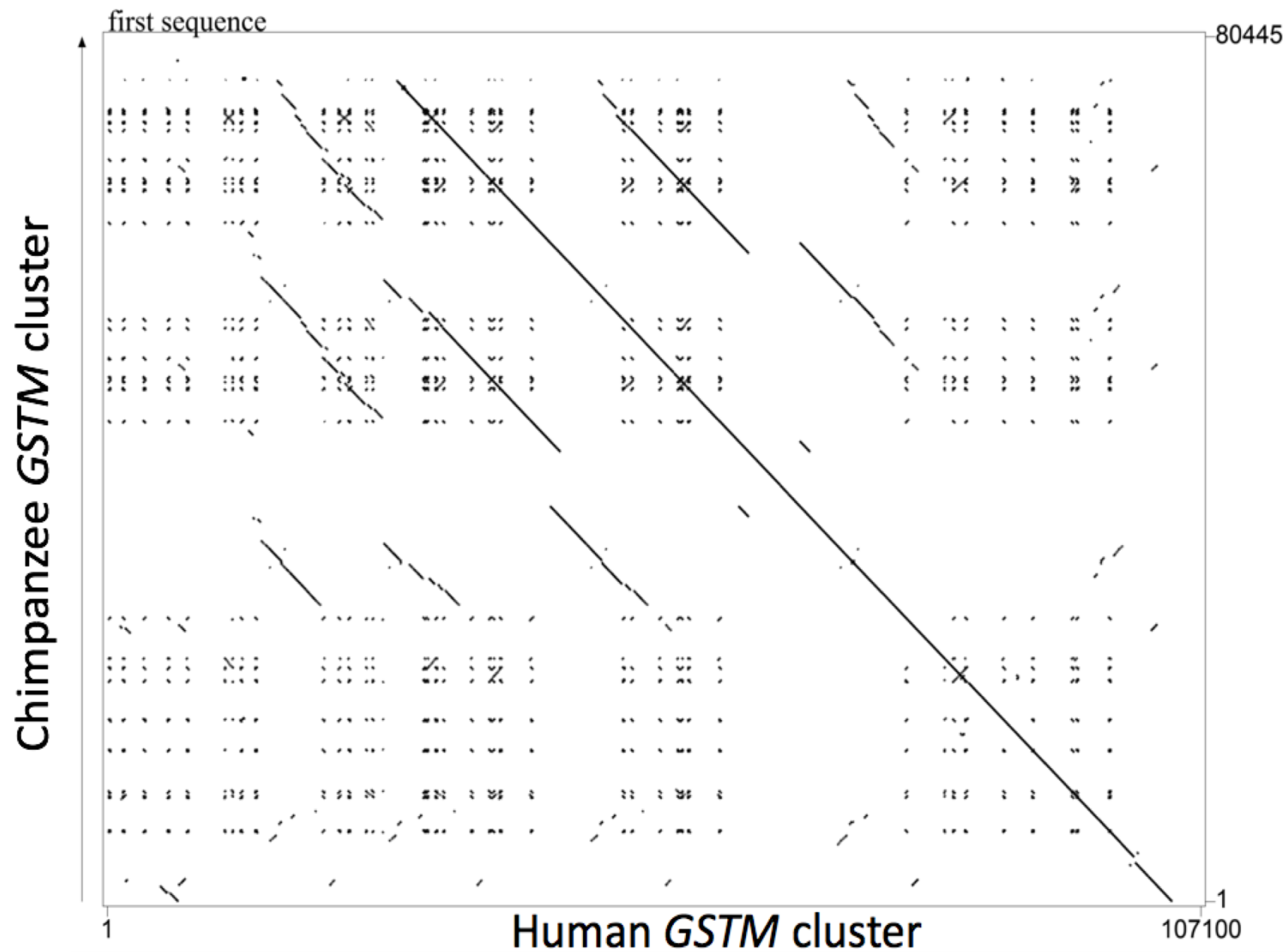

Supplement: Supplementary file 1 — Table S1. The functional GSTM analyzed. Table S2. The GSTM pseudogenes detected. Table S3. PCR primers and sequencing primers. Figure S1. NJ and ML tree of the primate and tree shrew GSTM genes. Figure S2. A dotplot of Humans and Chimpanzees GSTM gene cluster. Figure S3. Genotyping results in chimpanzees by ddPCR and read-depth approaches. Figure S4. The length and frequency of the deletions shared between humans and chimpanzees. Figure S5. The manual alignment of the SDs of humans, chimpanzees and orangutans. Figure S6. Maximum likelihood trees of the human and chimpanzee segmental duplications using MAFFT [66]. Figure S7. The probability of a deletion with a similar upstream breakpoint to recurrently evolve independently in chimpanzees and humans given a particular mutation rate. (ZIP 2977 kb) [file 12864_2018_4676_MOESM1_ESM.zip › Figure_S2.pdf]

(A) NJ tree

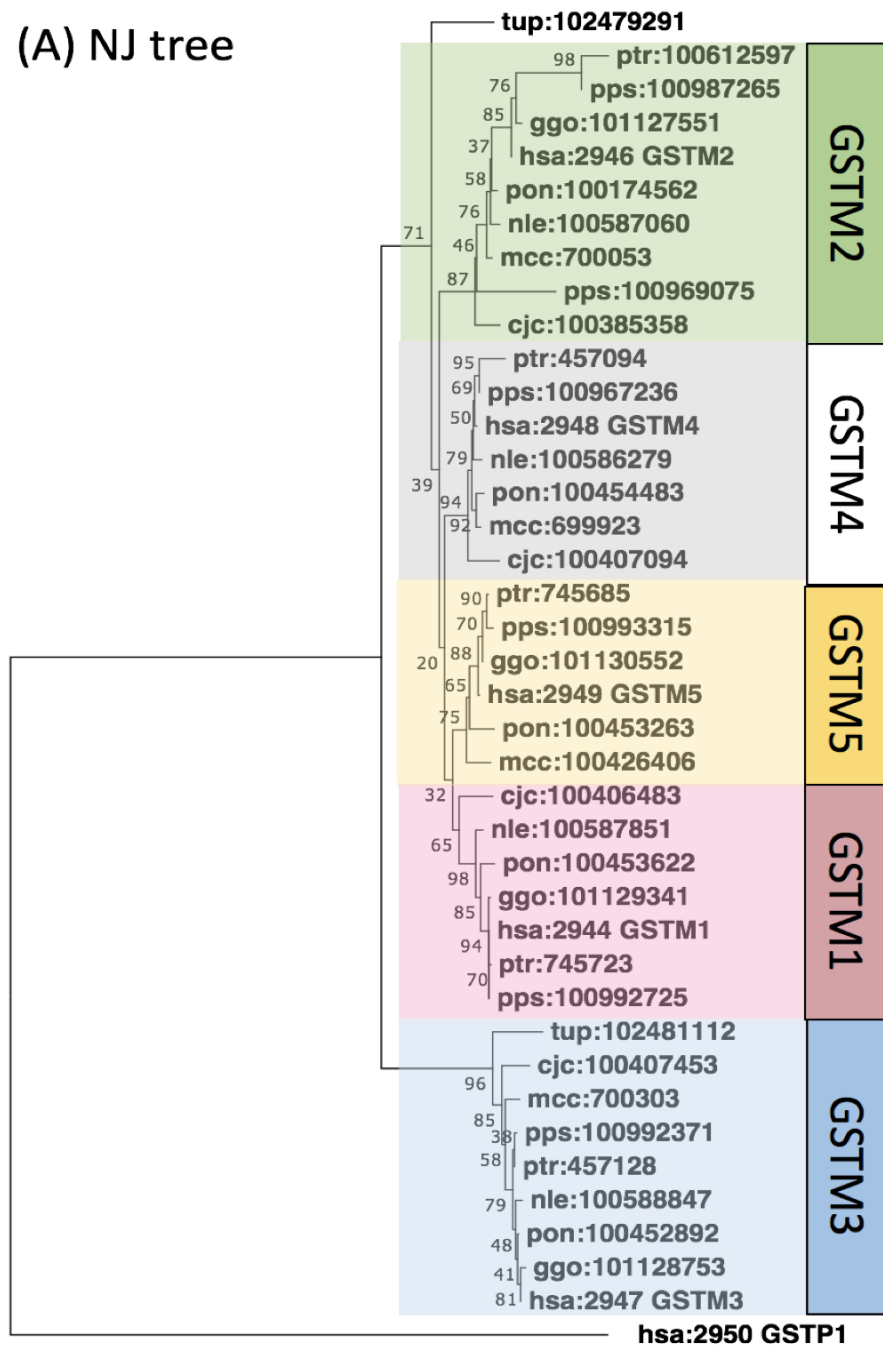

(B) ML tree

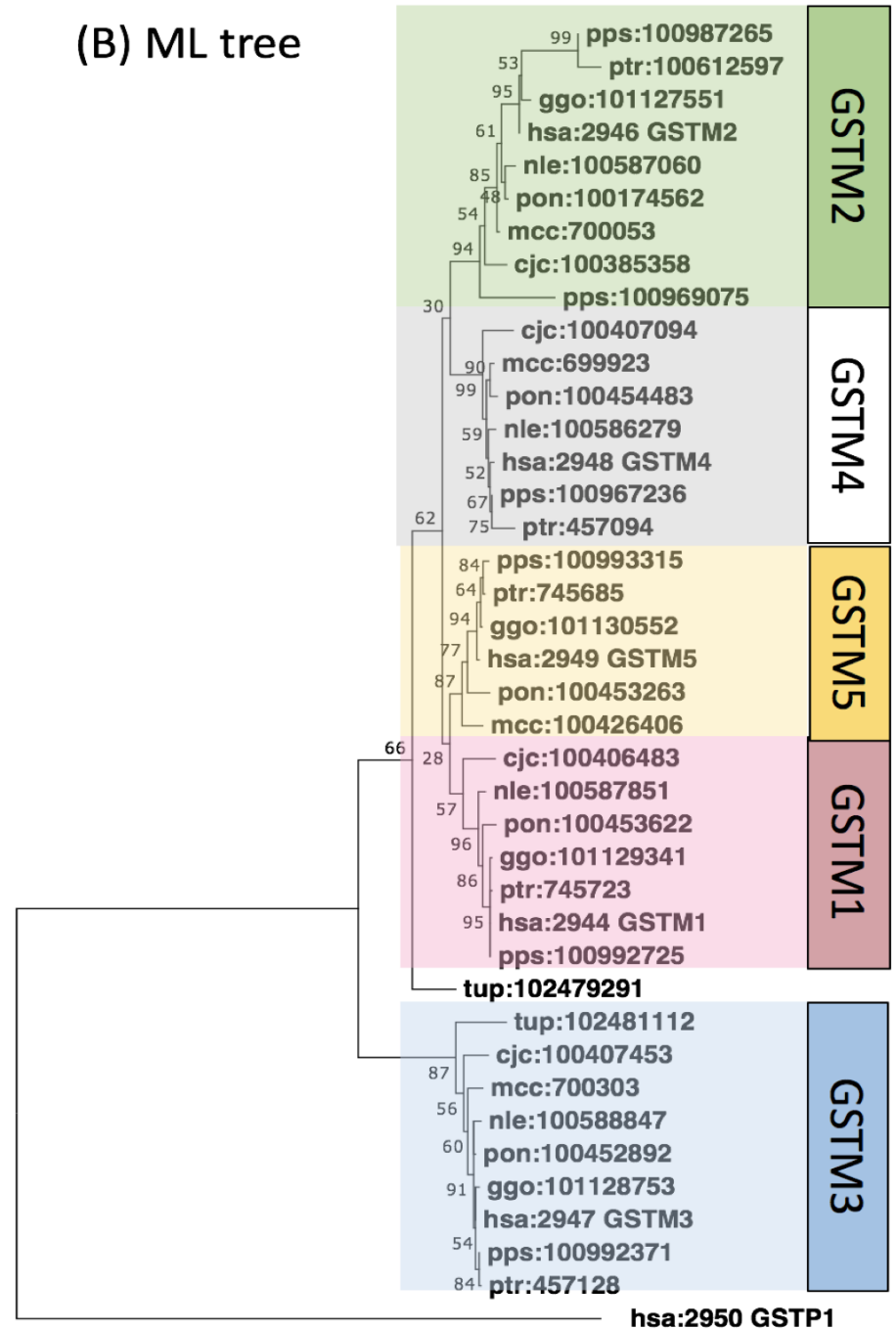

Supplement: Supplementary file 1 — Table S1. The functional GSTM analyzed. Table S2. The GSTM pseudogenes detected. Table S3. PCR primers and sequencing primers. Figure S1. NJ and ML tree of the primate and tree shrew GSTM genes. Figure S2. A dotplot of Humans and Chimpanzees GSTM gene cluster. Figure S3. Genotyping results in chimpanzees by ddPCR and read-depth approaches. Figure S4. The length and frequency of the deletions shared between humans and chimpanzees. Figure S5. The manual alignment of the SDs of humans, chimpanzees and orangutans. Figure S6. Maximum likelihood trees of the human and chimpanzee segmental duplications using MAFFT [66]. Figure S7. The probability of a deletion with a similar upstream breakpoint to recurrently evolve independently in chimpanzees and humans given a particular mutation rate. (ZIP 2977 kb) [file 12864_2018_4676_MOESM1_ESM.zip › Figure_S1.pdf]
