# Supplementary material for: Complex evolution of the GSTM gene family involves sharing of GSTM1 deletion polymorphism in humans and chimpanzees
Source: BMC Genomics. 2018 Apr 25;19:293. doi: 10.1186/s12864-018-4676-z (PMC5918908; doi:10.1186/s12864-018-4676-z)
Supplement: Supplementary file 1 — Table S1. The functional GSTM analyzed. Table S2. The GSTM pseudogenes detected. Table S3. PCR primers and sequencing primers. Figure S1. NJ and ML tree of the primate and tree shrew GSTM genes. Figure S2. A dotplot of Humans and Chimpanzees GSTM gene cluster. Figure S3. Genotyping results in chimpanzees by ddPCR and read-depth approaches. Figure S4. The length and frequency of the deletions shared between humans and chimpanzees. Figure S5. The manual alignment of the SDs of humans, chimpanzees and orangutans. Figure S6. Maximum likelihood trees of the human and chimpanzee segmental duplications using MAFFT [66]. Figure S7. The probability of a deletion with a similar upstream breakpoint to recurrently evolve independently in chimpanzees and humans given a particular mutation rate. (ZIP 2977 kb) [file Figure_S4.pdf › Figure_S4.pdf]

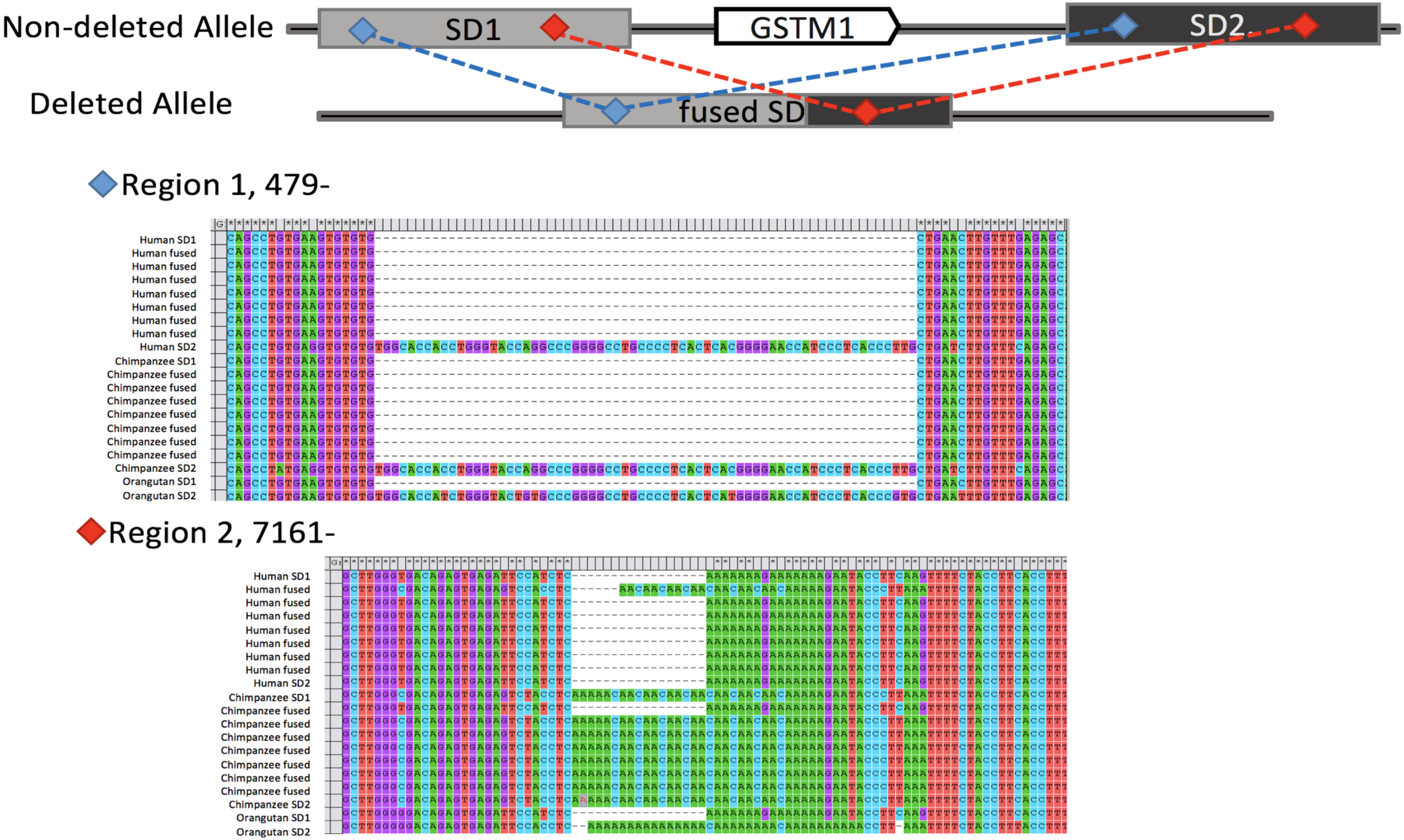
Non-deleted Allele
SD1
GSTM1
SD2
Deleted Allele
fused SD
Region 1, 479-
Human SD1
Human fused
Human fused
Human fused
Human fused
Human fused
Human fused
Human fused
Human SD2
Chimpanzee SD1
Chimpanzee fused
Chimpanzee fused
Chimpanzee fused
Chimpanzee fused
Chimpanzee fused
Chimpanzee fused
Chimpanzee fused
Chimpanzee SD2
Orangutan SD1
Orangutan SD2
Region 2, 7161-
Human SD1
Human fused
Human fused
Human fused
Human fused
Human fused
Human fused
Human fused
Human SD2
Chimpanzee SD1
Chimpanzee fused
Chimpanzee fused
Chimpanzee fused
Chimpanzee fused
Chimpanzee fused
Chimpanzee fused
Chimpanzee fused
Chimpanzee SD2
Orangutan SD1
Orangutan SD2
